# Different Growth and Physiological Responses to Cadmium of the Three *Miscanthus* Species

**DOI:** 10.1371/journal.pone.0153475

**Published:** 2016-04-12

**Authors:** Haipeng Guo, Chuntao Hong, Xiaomin Chen, Yanxia Xu, Yan Liu, Dean Jiang, Bingsong Zheng

**Affiliations:** 1 State Key Laboratory of Plant Physiology and Biochemistry, College of Life Sciences, Zhejiang University, Hangzhou, China; 2 Academy of Agricultural Sciences of Ningbo City, Ningbo, China; 3 Nurturing Station for the State Key Laboratory of Subtropical Silviculture, Zhejiang A & F University, Linan, Hangzhou, China; Wageningen University, NETHERLANDS

## Abstract

*Miscanthus* has been proposed as a promising crop for phytoremediation due to its high biomass yield and remarkable adaptability to different environments. However, little is known about the resistance of *Miscanthus* spp. to cadmium (Cd). To determine any differences in resistance of *Miscanthus* to Cd, we examined plant growth, net photosynthetic rate (Pn), activities of anti-oxidant and C_4_ photosynthetic enzymes, concentrations of Cd in leaves and roots, and observed the chloroplast structure in three *Miscanthus* species treated with 0, 10, 50, 100 or 200 μM Cd in solutions. *Miscanthus sinensis* showed more sensitivity to Cd, including sharp decreases in growth, Pn, PEPC activity and damage to chloroplast structure, and the highest H_2_O_2_ and Cd concentrations in leaves and roots after Cd treatments. *Miscanthus sacchariflorus* showed higher resistance to Cd and better growth, had the highest Pn and phosphoenolpyruvate carboxylase (PEPC) activities and integrative chloroplast structure and the lowest hydrogen peroxide (H_2_O_2_) and leaf and root Cd concentrations. The results could play an important role in understanding the mechanisms of Cd tolerance in plants and in application of phytoremediation.

## Introduction

Soil cadmium (Cd) pollution has posed a serious threat to our soil quality and food security as well as to human health. The sources of Cd contamination is not only introduced through geogenic processes but also derive from anthropogenic activities, such as the by-product of smelting, mining and refining of metal works [1], industrial waste from electroplating, manufacturing of plastics and paint pigments processes [2] and agriculture pollutions including impurities of fertilizers and irrigation with wastewater [3,4]. The toxicant of Cd is higher than that of organic toxic compounds due to its greater mobility and harder degraded and thus resulting in difficult to remove from the environment [5].

Cd is not a necessary element for plant growth and excess Cd has a series of harmful effects. Cd is known to inhibit plant growth, disorder nutrient uptake, affect chloroplast ultrastructure, inactivate enzymes of carbon dioxide (CO_2_) fixation, inhibit photosynthesis and damage the structure and function of photosystem II [6–8]. It was also reported that Cd generates oxidative stress in plants through inducing the production of reactive oxygen species (ROS), including superoxide anion radicals (O_2_^–^), hydroxyl radicals and hydrogen peroxide (H_2_O_2_) [9]. To remove ROS, plants have evolved a series of anti-oxidant enzymatic systems including superoxide dismutase (SOD), peroxidase (POD), catalase (CAT), ascorbate peroxidase (APX) and glutathione reductase (GR) [6]. Fernández et al. [7] showed that the effective Cd detoxification of *Dittrichia viscosa* (L.) Greuter consisted mainly of promoted APX activity and greater efficiency of CAT and GR.

Among the various strategies adopted for removal of Cd from contaminated soils, phytoremediation has been proposed as an economical, eco-friendly and aesthetically acceptable technology to reduce the risk of soil contamination [10,11]. The hyper-accumulation of heavy metals in some plants has been recorded by many researchers during the last few decades [12,13]. However, many hyper-accumulator plants have slow growth and low biomass, and are difficult to grow and harvest [14]. *Miscanthus* spp. has been proposed as promising crops for phytoremediation due to high biomass yield and remarkable adaptability to different environments [15,16]. *M*. *sinensis*, *M*. *floridulus* and *M*. *sacchariflorus* are all generally found along river banks, in mountain regions and open waste areas of China. *M*. *× giganteus* is widely cultured in Europe as a bio-energy crop, which is a sterile, triploid interspecific hybrid from native across with *M*. *sinensis* (diploid) and *M*. *sacchariflorus* (tetraploid) [17,18]. It has been reported that *Miscanthus sinensis* exhibits high resistance to the metal stress of acid soil by excreting citric acid [19]. *Miscanthus× giganteus* is tolerant not only to chromium (Cr), but also to Cd at certain concentration [20]. Pavel et al. [21] reported that *M*. *sinensis* × *giganteus* be used for the production of renewable biomass on zinc (Zn), Cd and lead (Pb) contaminated soils, and for further increase in biomass and reduction of the metal concentrations of plant tissues upon addition of red mud to these soils. However, the related research on the responses of *M*. *floridulus* and *M*. *sacchariflorus* to Cd stress is scarce. Moreover, there is a lack of data on Cd tolerance and comparisons among different *Miscanthus* spp. to evaluate their capacity for phytoremediation. Thus, it is of importance, for removing Cd from contaminated soils, to elucidate the mechanism of Cd resistance of different *Miscanthus* spp.

## Materials and Methods

### Ethics statement

The seeds of *M*. *sinensis*, *M*. *floridulus* and *M*. *sacchariflorus* were obtained from the Daming Mountain Scenic Area in Linan, Zhejing Province of China, in 2013. The three *Miscanthus* species are widely distributed throughout this Scenic Area and the local government departments have no special requirements to protect them. The experiments were also permitted according to the rules of Zhejiang University. So these will not cause any controversy. It is also confirmed that the field studies did not involve endangered or protected species by the institute of plant science, Zhejiang University.

### Plant materials and growth conditions

Mature seeds of *M*. *sinensis*, *M*. *floridulus* and *M*. *sacchariflorus* were planted in commercial potting mix in plastic trays, and then allowed to germinate at 28°C in the dark for 3 d. Four weeks after germination, the seedlings were transferred to hydroponic cultures supplied with half-strength Hoagland nutrient solution (pH 6.0). Half-strength Hoagland nutrient solution was used containing the following macronutrients in mM: KNO_3_, 2.5; Ca(NO_3_)_2_·4H_2_O, 2.5; MgSO_4_·7H_2_O, 1.0; NH_4_H_2_PO_4_, 0.5, and the following micronutrients in μM: CuSO_4_·5H_2_O, 0.5; ZnSO_4_·7H_2_O, 1.0; MnCl_2_, 1.25; H_3_BO_3_, 7.5; (NH_4_)_6_Mo_7_O_24_, 0.25 and NaFeEDTA 50. To ensure proper growth, the solutions were aerated and renewed weekly. Following 32 days of hydroponic growth, seedlings were subjected to aerated nutrient solution including 0, 10, 50, 100 or 200 μM CdCl_2_. Each treatment was replicated six times and each replicate included eight seedlings. The solutions were renewed every week. The entire experiment was conducted in an environmentally controlled growth room with a 14 h/26°C day (white fluorescent light intensity of 1200 μmol photons m^−2^ s^−1^) and 10 h/22°C night regime with relative humidity kept at 65%.

### Growth analysis and Cd contents measurement

Growth such as plant height, root length, aerial part and hypogeal-part dry weight were measured after 16 d of treatment. Plant height and the length of the below ground part (root length) were measured on a centimeter scale. Dry weight was determined after drying the samples in an oven at 80°C to a constant weight. The root:shoot ratio was computed as the hypogeal part divided by the aerial part on a dry weight basis.

For Cd content measurement, roots and shoots were separately harvested, and the roots were washed with deionized water for three times. Then shoots and roots were dried at 80°C for 72 h, weighed, ground to fine powder and 0.2 g of each was digested with nitric acid/H_2_O_2_ (30:1, v/v) and total Cd content was measured by inductively coupled plasma atomic emission spectrometer (ICP-AES; Fisons ARL Accuris, Ecublens, Switzerland).

### Determination of photosynthetic and chlorophyll fluorescence parameters

Photosynthetic parameters of leaves were measured with a Li-6400 portable photosynthesis system (LI-COR, Lincoln, NE, USA). These parameters consisted of net photosynthetic rate (Pn), stomata conductance (g_s_) and intercellular CO_2_ concentration (Ci). The data were recorded at 16 d of treatment using the most fully expanded youngest leaves. The light intensities were maintained at 2000 μmol m^–2^ s^–1^, and the temperature and external CO_2_ concentration were maintained at 30°C and 400 μmol L^–1^, respectively. Five representative plants of each treatment were selected randomly at each measured time-point. For light response curves measurements, a series of light intensities were set as 2500, 2000, 1500, 1200, 800, 600, 400, 300, 200, 100, 50, 30, 10, 0 μmol m^–2^ s^–1^ PPFD at an ambient CO_2_ concentration 400 μmol mol^–1^ with the *LI-COR* CO_2_ mixer. Minimum time and maximum time were respectively set to 1min and 2 min for each given PPFD. Before the measurement, each leaf was adapted at a PPFD of 2500 μmol m^–2^ s^–1^ for about 5 min until the stability state of Pn. According to the modified rectangular hyperbola model[22], light compensation point (LCP), the maximum photosynthetic rate (Pn_MAX_), apparent quantum yield (AQY) and dark respiratory rate (DR) were calculated as: *P(I)* = α·*I·(1-*β·*I)/(1+*γ·*I)-R*_*d*_. Where *P(I)* is Pn, *I* is light intensity, *R*_*d*_ is dark respiratory rate, and α, β and γ are coefficients which are independent of *I*. Once Pn was obtained, the leaf tissue was freeze-clamped quickly at liquid N_2_ temperature and stored at –80°C for chlorophyll, malondialdehyde (MDA), hydrogen peroxide (H_2_O_2_) contents and enzyme activity analysis.

The chlorophyll fluorescence parameters were measured with an chlorophyll fluorescence imaging system (*CF imager*, Technologica Ltd., Colchester, UK) according to the method of Liu et al. [23] with minor modification. The first fully grown leaves of *Miscanthus* seedlings treated with different concentrations of Cd were dark-adapted for 20 min with leaf clips, then the leaves were cut off and arranged neatly underneath the fluorometer for recording the minimum fluorescence (*F*_*0*_) and maximum fluorescence (*Fm*) parameters and getting the false-color images of maximal photochemical efficiency (*Fv/Fm*) images. The *Fv/Fm* was calculated as *(Fm- F*_*0*_*)/Fm*. Then leaves were light-adapted for approximately 15 min prior to measurement of the effective PSII quantum yield [Y(II)] which was calculated as Y(II) = *(Fm'-F)/Fm'*, where *Fm'* and F were fluorescence at maximum fluorescence and steady-state photosynthesis in the light, respectively.

### Determination of photosynthetic pigment contents

Photosynthetic pigments were extracted by soaking 0.1 g of frozen leaf tissues in 80% (v/v) acetone in darkness at room temperature for 45 h. Chlorophyll and carotenoid contents in supernatants were determined with a spectrophotometer (UV-2550, Shimadzu, Kyoto, Japan) at 665, 649 and 470 nm, and calculated using the method of Lichtenthaler and Wellburn [24].

### Determination of C_4_ photosynthetic enzyme activities

The phosphoenolpyruvate carboxylase (PEPC), NADP-malate enzyme (NADP-ME) and NADP-malate dehydrogenase (NADP-MDH) activities of leaves were determined using a commercial chemical assay kit (Jiangsu Keming Biotechnology Institute, Suzhou, China). For the measurement of PEPC and NAD-MDH activities, about 0.1 g of frozen leaf tissues were homogenized in 1 ml buffer I [0.4 M Tris-HCl buffer (pH 8.0), 15 mM EDTA, 10 mM DTT, 5 mM MgCl_2_ and 2% (w/v) polyethylene pyrrole (PVP)], which is contained in the commercial chemical assay kit, at 4°C with an ice-chilled pestle and mortar, centrifuged at 10,000 rpm at 4°C for 10 min and the supernatant was used for the enzymes activity analysis according to the manufacturer’s instructions. For the measurement of NADP-ME activity, about 0.1 g of frozen leaf tissues were extracted using 1 ml buffer I [0.1 mM KH_2_PO_4_/KOH buffer (pH 7.5), 10 mM DTT, 5 mM MgCl_2_ and 2% (w/v) polyethylene pyrrole (PVP)] according to the above-mentioned method, then analyzed according to the manufacturer’s instructions.

### Chloroplast ultrastructure

The chloroplast ultrastructure of bundle sheath cells were observed according to Shao et al. [25]. After 16 days treatment, the fully expanded youngest leaves were immediately fixed in 2.5% (v/v) glutaraldehyde (0.1 mol L^–1^ phosphate buffer, pH 7.2) for 24 h. Then the samples were post-fixed for 30 min in 1% (v/v) osmium acid, dehydrated in a graded ethanol series (30%–100%, v/v), embedded in Spurr resin and ultrathin-sectioned for transmission electron microscopy (H7650, Hitachi, Tokyo, Japan).

### Determination of MDA, H_2_O_2_ contents and anti-oxidant enzymes activities

For the determination of SOD, CAT and POD activities, about 0.5 g of frozen leaf tissues were ground at 4°C in a mortar with 5 ml of 50 mM phosphate buffer solution (pH 7.8) containing 1% PVP. The homogenate was centrifuged at 10,000 rpm at 4°C for 30 min. Supernatants were collected for measuring enzyme activities according to Hong et al. [26]. The MDA, H_2_O_2_ contents, GR and APX activities, were determined using a commercial chemical assay kit (Jiangsu Keming Biotechnology Institute, Suzhou, China). For the measurement of MDA content and GR and APX activities, about 0.1 g of frozen leaf tissues were homogenized in 1 ml buffer I [50 mM phosphate buffer (pH 7.8), containing 0.1 mM EDTA, 0.5% (w/v) Triton-100 and 2% PVP], which is contained in the commercial chemical assay kit, at 4°C with mortar and pestles, centrifuged at 10,000 rpm at 4°C for 10 min and the supernatant was used for content or enzyme ability analysis according to the manufacturer’s instructions. For the measurement of H_2_O_2_ contents, the extraction buffer I was replaced by 1 ml acetone according to the above-mentioned method, then analyzed according to the manufacturer’s instructions.

### Statistical analysis

Statistical analysis was carried out by one-way or two-way analysis of variance using SPSS (SPSS Inc., USA, version 13.0) and OriginPro (OriginLab Corp., USA, v8.0724). Differences between treatments were evaluated at P < 0.05.

## Results

### Plant growth and Cd accumulation

**Fig 1:**
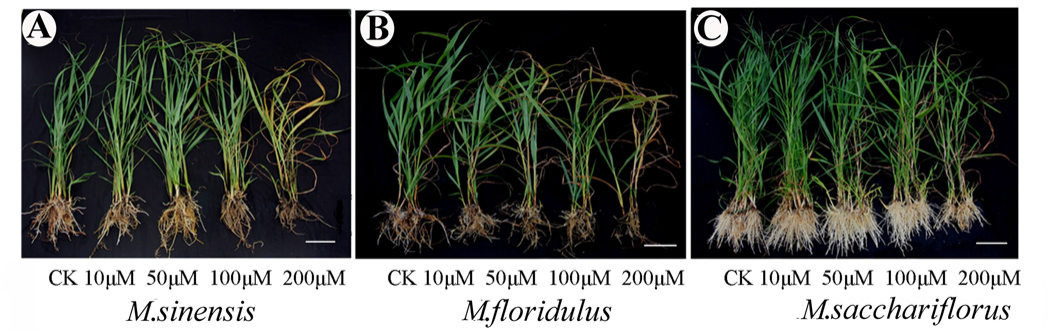
Effects of different Cd treatments on seedling growth and morphology of three *Miscanthus* species after 16 days of Cd treatment. Growth characteristics of seedlings of (A) *M*. *sinensis*, (B) *M*. *floridulus* and (C) *M*. *sacchariflorus*. *Miscanthus* seedlings (60-d-old) were grown in Hoagland nutrient solution (pH 6.0) containing 0, 10, 50, 100 and 200 μM CdCl_2_ for 16 days, respectively. Bar = 10 cm.

The sensitivities of *Miscanthus* spp. to Cd varies, and roots were more sensitive than shoots (Fig 1 and Table 1). The growth parameters such as root length, dry weight of the hypogeal part and the entire plant dry weight were significantly inhibited (*p<0*.*05*) by all Cd concentrations and plant height was significantly decreased (*p<0*.*05*) by ≥ 50 μM Cd concentrations in *Miscanthus sinensis* (Table 1). For *M*. *floridulus* there were no significant differences (*p<0*.*05*) in root length between 10 μM Cd treatment and control (0 μM Cd), and no significant differences (*p<0*.*05*) in plant height, hypogeal part and entire plant between 10 and 50 μM Cd; however, there was a significant difference (*p<0*.*05*) in dry weight of aerial parts between all Cd treatments. For *M*. *sacchariflorus*, however, in comparison with control 10 μM Cd treatment slightly promoted plant growth according to all growth indexes (Table 1) and 50 μM Cd treatment significantly increased (*p<0*.*05*) in dry weight of the hypogeal part and the entire plant, and in root:shoot ratio. Therefore, *M*. *sacchariflorus* was more resistant to Cd than the other *Miscanthus* spp.

**Table 1:** Growth and dry weight of three *Miscanthus* species in response to Cd stress for 16 days. Values represent mean ± SD (n = 4). Differences letters indicate significant differences (P < 0.05) between Cd levels.

Cultivars	Cadmium (μM)	Plant height (cm)	Root length (cm)	Dry weight (g per plant)			Root-shoot ratio
				Aerial part	Hypogeal part	Entire plant	
*M*. *sinensis*	0	89.33±1.53a	23.00±1.73a	4.06±0.09a	1.10±0.09a	5.16±0.04a	0.271±0.021a
	10	87.67±2.08a	20.17±0.76b	3.96±0.11a	0.89±0.05b	4.85±0.16b	0.225±0.020c
	50	85.12±1.52b	16.67±0.58c	3.72±0.08b	0.82±0.01c	4.54±0.20c	0.220±0.014d
	100	77.33±1.53c	14.37±0.58d	3.17±0.07c	0.74±0.01d	3.91±0.18d	0.233±0.020b
	200	73.00±2.65d	12.00±1.00e	2.44±0.25d	0.57±0.03e	3.02±0.22e	0.234±0.010b
*M*. *floridulus*	0	87.23±1.37a	16.00±1.00a	3.47±0.36a	0.81±0.04a	4.28±0.07a	0.232±0.010c
	10	83.57±2.04b	15.33±0.58a	3.08±0.17b	0.72±0.07b	3.80±0.19b	0.234±0.030c
	50	82.00±1.73b	14.17±0.76b	2.98±0.07c	0.70±0.04b	3.68±0.22b	0.235±0.017c
	100	76.38±1.53c	13.13±1.21c	2.68±0.04d	0.65±0.03c	3.33±0.04c	0.243±0.024b
	200	72.33±1.53d	11.93±0.90d	2.15±0.13e	0.55±0.09d	2.70±0.07d	0.256±0.033a
*M*. *Sacchariflorus*	0	93.33±1.15a	17.67±1.53a	5.41±1.16a	1.52±0.32b	6.93±1.45b	0.281±0.029d
	10	94.56±1.03a	18.00±1.00a	5.72±1.18a	1.65±0.35b	7.37±1.92a	0.288±0.028d
	50	91.63±1.53a	18.12±1.26a	5.20±0.74a	2.25±0.18a	7.45±1.12a	0.432±0.010a
	100	86.78±1.53b	15.33±1.15b	4.60±0.09b	1.82±0.26b	6.42±0.18c	0.396±0.064b
	200	82.16±2.08c	10.50±0.50c	2.97±0.29c	0.99±0.12c	3.96±0.36d	0.333±0.037c

**Fig 2:**
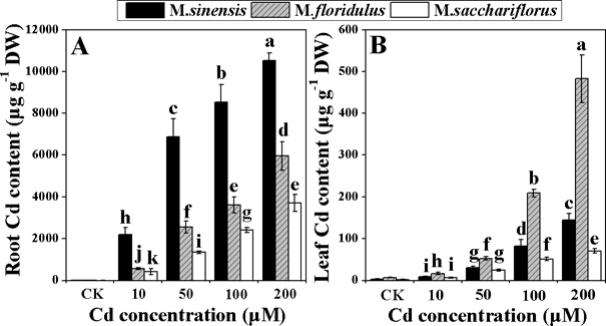
**Change in root Cd content (A) and leaf Cd content (B).** Data are mean ± SD (n = 3). Different letters indicate a significant difference at P < 0.05.

The Cd contents of roots and leaves of *Miscanthus* spp. were extremely different, although they significantly increased (*p<0*.*05*) with increasing Cd concentration (Fig 2A and 2B). In roots, *M*. *sinensis* exhibited the highest Cd concentration, followed by *M*. *floridulus* and then *M*. *sacchariflorus* (Fig 2A). In leaves, *M*. *floridulus* had the highest Cd concentration, then *M*. *sinensis* and *M*. *sacchariflorus* (Fig 2B). Under 200 μM Cd treatment, Cd concentrations in leaves of *M*. *sinensis*, *M*. *floridulus* and *M*. *sacchariflorus* were 146, 210 and 71 μg g^–1^ dry weight, respectively, while correspondingly in roots they were 10.55, 5.96 and 3.72 mg g^–1^ dry weight. These results suggested that the Cd mainly accumulated in roots in *M*. *sinensis* and was transported to leaves in *M*. *floridulus*, while *M*. *sacchariflorus* accumulate less Cd in total.

### Photosynthesis

**Fig 3:**
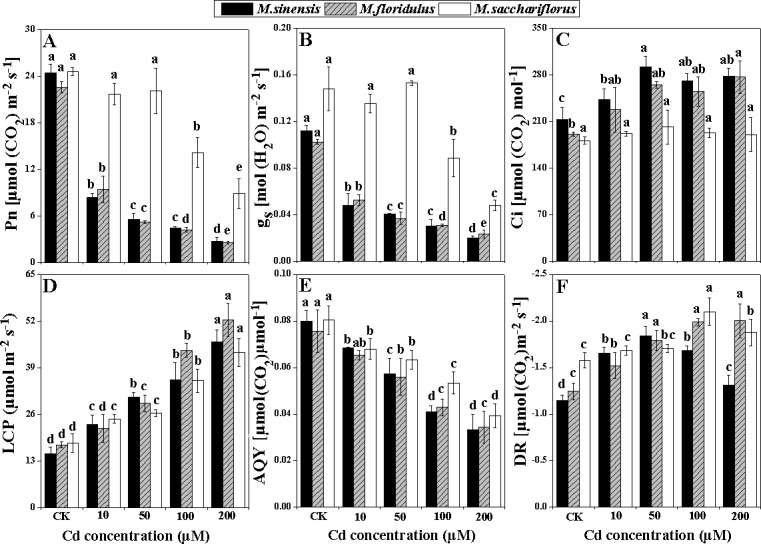
Changes in photosynthetic parameters. (A) Net photosynthetic rate (Pn), (B) stomata conductance (g_s_), (C) intercellular CO_2_ concentration (Ci), (D) light compensation point (LCP), (E) apparent quantum yield (AQY), (F) dark respiratory rate (DR). Data are mean ± SD (n = 3). Different letters indicate a significant difference at P < 0.05.

Photosynthetic parameters among the three *Miscanthus* species significantly differed (*p<0*.*05*) with increasing exogenous Cd concentrations (Fig 3). When treated with 10 μM Cd, Pn and g_s_ of *M*. *sinensis* and *M*. *floridulus* were significantly depressed (*p<0*.*05*) to about one-third of their control values and continuously decreased to 10–20% of controls under 200 μM Cd treatment. However, there were no significant decreases (*p<0*.*05*) in Pn and g_s_ of *M*. *sacchariflorus* for Cd concentrations < 50 μM, and it maintained about one-third of Pn and g_s_ of controls even when treated with 200 μM Cd (Fig 3A and 3B). Ci enhanced with increasing Cd concentrations for *M*. *sinensis* and *M*. *floridulus*, but did not change for *M*. *sacchariflorus* (Fig 3C). Pn in three *Miscanthus* species also decreased significantly with increasing Cd concentrations in different light conditions (S1 Fig). Under PAR of 2500 μmol m^-2^ s^-1^, the Pn of *M*. *sinensis* was decreased by 10.7, 61, 76.9 and 83% in the 10, 50, 100 and 200 μM Cd treatments, respectively (S1A Fig); it was decreased by 36.6, 53.7, 57.4 and 85.1% in *M*. *floridulus* (S1B Fig) and 10, 38.7, 54.9 and 84.3% in *M*. *sacchariflorus*, respectively (S1C Fig). Similar trends for the effect of different Cd concentrations on the gs of the three *Miscanthus* species were observed (S1D–S1F Fig) in different light conditions. According to the light response curve (S1 Fig) it was observed that LCP sharply raised (Fig 3D) and AQY dramatically decreased (*p<0*.*05*) in all species with increased Cd concentrations (Fig 3E), but the degree of increase in LCP and decrease in AQY was least in *M*. *sacchariflorus* (Fig 3D and 3E). Moreover, DR gradually increased and reached a maximum for 50–100 μM Cd treatments in all *Miscanthus* spp. (Fig 3F).

### Photosynthetic pigment contents

**Fig 4:**
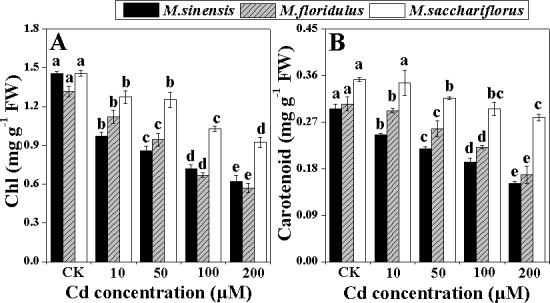
Change in photosynthetic pigments content. (A) Chlorophyll (Ch) and (B) carotenoid. Data are mean ± SD (n = 3). Different letters indicate a significant difference at P < 0.05.

Chlorophyll and carotenoid contents were significantly decreased (*p<0*.*05*) by increased Cd concentrations for all *Miscanthus* spp., and the reductions were always lower in *M*. *sacchariflorus* than for the other species (Fig 4). *Miscanthus sinensis* and *M*. *floridulus* showed similar decreases in chlorophyll and carotenoid contents, especially at 100 and 200 μM Cd treatments. The chlorophyll contents and carotenoid contents of *M*. *sinensis* decreased by 57.7% and 48.6%, respectively and had corresponding decreases in *M*. *floridulus* of 56.8% and 44.8% in comparison to control, whereas 36.6% and 20.8% reduction was noted in *M*. *sacchariflorus* under 200 μM Cd stress (Fig 4A and 4B).

### C4 photosynthetic enzymes activities and Chloroplast structure

**Fig 5:**
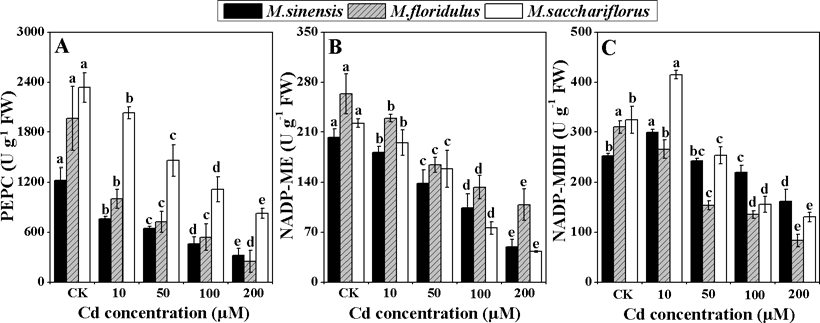
Change in C4 photosynthetic enzymes activities. (A) Phosphoenolpyruvate carboxylase (PEPC), (B) NADP-malate enzyme (NADP-ME), (C) NADP-malate dehydrogenase (NADP-MDH). Data are mean ± SD (n = 3). Different letters indicate a significant difference at P < 0.05.

PEPC activity differed markedly between the *Miscanthus* spp., although it significantly decreased (*p<0*.*05*) under Cd stress. The highest activity was in *M*. *sacchariflorus*, whereas the lowest in *M*. *sinensis* (Fig 5A). The inhibitory effect of Cd on PEPC activity was more evident for *M*. *sinensis* and *M*. *floridulus* than for *M*. *sacchariflorus*. The PEPC activities were decreased by 37.9%, 46.9%, 62.5% and 74.5% in *M*. *sinensis*, respectively and 49.1%, 63.0%, 72.5% and 87.2% in *M*. *floridulus*, respectively, in comparison to control, whereas 13.0%, 37.5%, 52.2% and 64.5% reduction was found in *M*. *sacchariflorus* with increasing Cd concentrations, respectively (Fig 5A). NADP-ME activity also decreased significantly (*p<0*.*05*) for all *Miscanthus* spp. under Cd stress with slightly higher activity for *M*. *floridulus* than the other species for all Cd concentrations (Fig 5B). The greatest reduction in NADP-ME activity was for 100 and 200 μM Cd treatments in *M*. *sacchariflorus*, with inhibition ratios of 68.1% and 81.6%, respectively (Fig 5B). Low concentration of exogenous Cd (10 μM) enhanced the NADP-MDH activity in *M*. *sinensis* and *M*. *sacchariflorus*, and all Cd concentrations resulted in significantly decreased (*p<0*.*05*) NADP-MDH activity in *M*. *floridulus* (Fig 5C). In addition, NADP-MDH activity was significantly inhibited (*p<0*.*05*) by 50, 100 and 200 μM Cd treatments in *M*. *sinensis* and *M*. *sacchariflorus*; resulting in reductions of 50.3%, 56.2% and 73.1% in *M*. *sinensis*, respectively; and correspondingly 22.1%, 52.0% and 59.7% in *M*. *sacchariflorus* (Fig 5C).

**Fig 6:**
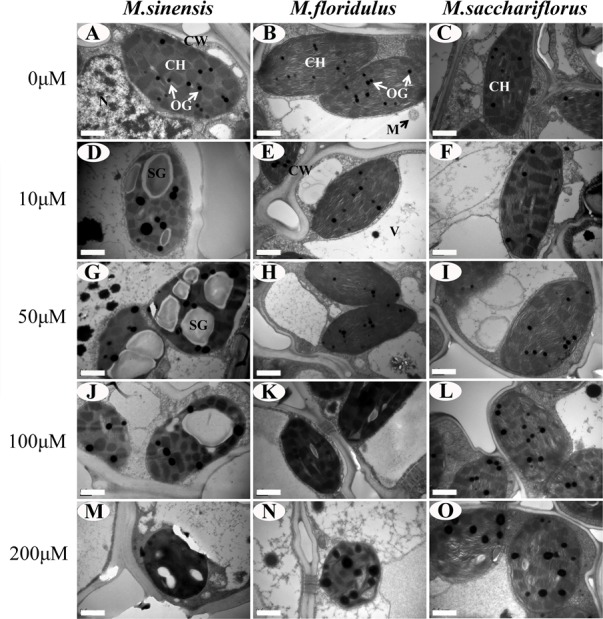
The chloroplast ultrastructure in bundle sheath cells. (A,D,G,J,M) *M*. *sinensis*; (B,E,H,K,N) *M*. *floridulus*; and (C,F,I,L,O) *M*. *sacchariflorus*. Note the differences in number of osmiophilic globules (OG, indicated by arrows) between different Cd treatments. Abbreviations: CH, chloroplast; CW, cell wall; SG, starch grains; M, mitochondria; V, vacuole.

The structural changes in chloroplasts markedly differed between *Miscanthus* spp. under Cd stress (Fig 6 and S2 Fig). With zero Cd treatment, chloroplasts in all species showed well-developed structures with normal granal and stromal thylakoids and some small osmiophilic globules (Fig 6A–6C). Treatment ≥ 10 μM Cd dramatically increased production of starch grains and enlarged osmiophilic globules in *M*. *sinensis* (Fig 6D, 6G and 6J); 100 μM Cd caused accumulation of small starch grains and enlargement of osmiophilic globules in *M*. *floridulus* (Fig 6K and 6N); but in *M*. *sacchariflorus*, only 200 μM Cd resulted in accumulation of small starch grains and enlargement osmiophilic globules (Fig 6L and 6O). The chloroplast envelope became indistinct in *M*. *sinensis* treated with ≥ 50 μM Cd (Fig 6G, 6J and 6M) and in *M*. *floridulus* treated with ≥ 100 μM Cd. Higher concentrations of exogenous Cd caused the granal and stromal lamellae of chloroplasts to condense and a loss of connection between both lamellae in *M sinensis* (Fig 6G, 6J and 6M) and *M*. *floridulus* (Fig 6N). The chloroplast structure in *M*. *sacchariflorus* did not change significantly for all Cd concentrations.

### Contents of MDA and H_2_O_2_

**Fig 7:**
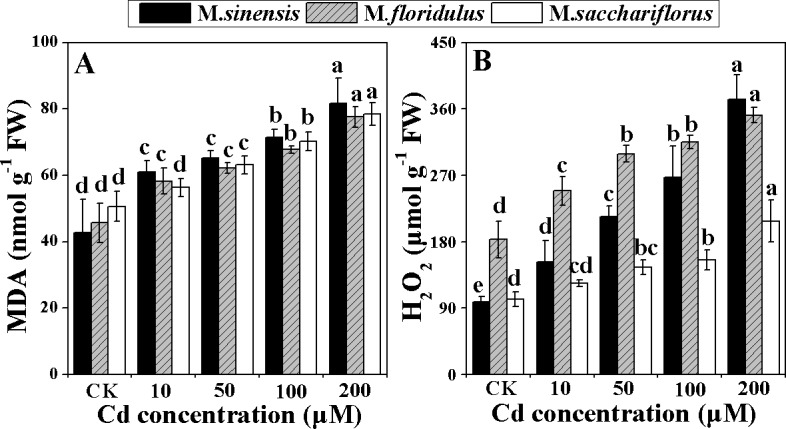
**Change in (A) malondialdehyde (MDA) and (B) hydrogen peroxide (**H_2_O_2_**) content.** Data are mean ± SD (n = 3). Different letters indicate a significant difference at P < 0.05.

The MDA accumulation increased in all the three species under Cd stress. The degrees of increment varied for the 10, 50, 100 and 200 μM Cd treatments, with 43%, 53%, 67% and 91% in *M*. *sinensis*, respectively; and correspondingly 29%, 37%, 48% and 64% in *M*. *floridulus*; and 11%, 25%, 39% and 55% in *M*. *sacchariflorus* (Fig 7A). The H_2_O_2_ contents also increased under Cd stress in all the three *Miscanthus* spp.. As shown in Fig 7B, they were the highest in *M*. *sinensis* with about three times the control values for 200 μM Cd and only about two times greater for both *M*. *floridulus* and *M*. *sacchariflorus* treated with 200 μM Cd.

### Activities of anti-oxidant enzymes

**Fig 8:**
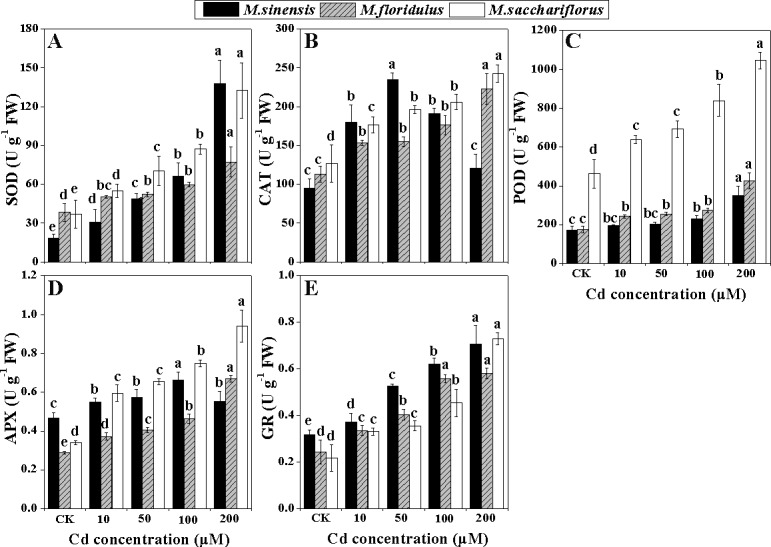
**Change in activities of antioxidant enzymes (A) Superoxide dismutase (SOD), (B) catalase (CAT), (C) peroxidase (POD), (D) ascorbate peroxidase (APX) and (E) glutathione reductase (GR).** Data are mean ± SD (n = 3). Different letters indicate a significant difference at P < 0.05.

All anti-oxidant enzymes including SOD, CAT, POD, APX and GR had markedly increased activity in leaves of *Miscanthus* spp. treated with Cd (Fig 8A–8E). SOD activity in leaves showed a higher increasing trend in *M*. *sinensis* and *M*. *sacchariflorus* than in *M*. *floridulus* under Cd stress. Compared to controls, 200 μM Cd treatment resulted in SOD activity of 7.5 times higher in *M*. *sinensis* and 3.6 times in *M*. *sacchariflorus* (Fig 8A). CAT activity in *M*. *sinensis* increased significantly (*p<0*.*05*) with Cd treatment up to 50 μM Cd, and then decreased with further increasing Cd concentrations; while in *M*. *floridulus* and *M*. *sacchariflorus*, CAT activity raised continuously with increasing Cd levels, to about twice the control values for 200 μM Cd treatment (Fig 8B). POD activities also varied among the species (Fig 8C) and were much higher in *M*. *sacchariflorus* than in *M*. *sinensis* and *M*. *floridulus*. Cd treatments significantly promoted (*p<0*.*05*) POD activities, especially in *M*. *sacchariflorus*, but only high Cd concentrations (100 and 200 μM) significantly up-regulated (*p<0*.*05*) POD activity in *M*. *floridulus*. APX activities in *M*. *sacchariflorus* were greatly enhanced by Cd treatments and reached a peak for *M*. *sinensis* at 100 μM Cd (Fig 8D). GR activity was lower in *M*. *sacchariflorus* than in *M*. *sinensis* and *M*. *floridulus* with increasing Cd concentrations, except for 200 μM Cd treatment where GR activity increased by 2.4, 1.2 and 1.4 times, respectively, compared with their controls (Fig 8E).

## Discussion

### Different response of growth and Pn in *Miscanthus* spp. to exogenous Cd concentrations

Cd is a trace pollutant that is toxic to plants, animals and humans. In the present study, all Cd levels negatively influenced plant growth of *M*. *sinensis* and *M*. *floridulus*, causing significant reductions (*p<0*.*05*) in plant growth and dry biomass, while, there was a slight increase in growth of *M*. *sacchariflorus* at < 50 μM Cd and lower degree of reduction compared to *M*. *sinensis* and *M*. *floridulus* at 200 μM Cd, suggesting that *M*. *sacchariflorus* had greater Cd stress tolerance than *M*. *sinensis* and *M*. *floridulus*. It has also been reported that plant genotypes differ in their tolerance to Cd toxicity [6]. At high Cd concentrations, the leaves of *M*. *sinensis* and *M*. *floridulus* became yellow and roots became soft and brown, while the leaves remained green and the roots white for *M*. *sacchariflorus*, even at 200 μM Cd (Fig 1), thus further confirming greater Cd tolerance of *M*. *sacchariflorus* among the three species. Arduini et al. [15] find that, even for long-term, low Cd (0.5 mg L^-1^) application stimulates *Miscanthus* growth. Gill et al. [27] reported that at 25 mg kg^-1^ soil Cd, co-ordination of S and N metabolism can still complement to the antioxidant mechanism to protect the growth and photosynthesis of *Lepidium sativum* plants. However, high Cd doses (50–100 μM) cause growth inhibition and even plant death owing to inhibiting photosynthesis, respiration, water and nutrient uptake [28,29].

The inhibitory effect of Cd on Pn, g_s_ and chlorophyll content was more evident in *M*. *sinensis* and *M*. *floridulus* than *M*. *sacchariflorus*. The growth inhibition may be a consequence of Cd interference with the main metabolic processes such as photosynthesis and translocation of photosynthetic products and nutrient elements [30]. In the present study, the decrease in whole plant dry weight was in accordance with the decrease of Pn (Fig 3A and Table 1), suggesting that Pn played an important role in biomass accumulation during Cd stress. The Cd-induced reduction in Pn and AQY (Fig 3A and 3E) could be partially due to the decrease in g_s_ and chlorophyll content of the *Miscanthus* species (Figs 3B and 4A), as reported for maize (*Zea mays* L.) [31, 32] and sugarcane (*Saccharum officinarum* L.) [33]. The mechanism of photosynthetic response involves both stomatal and non-stomatal effects under environmental stress in C_4_ crops [34–36]. The results showed that the decrease in Pn was accompanied by increasing Ci concentration in *M*. *sinensis* and *M*. *floridulus*, suggesting that the factor limiting photosynthesis was mainly non-stomatal under Cd stress [34–37]. However, such changes were absent in *M*. *sacchariflorus*, implying different mechanisms for Pn depression due to Cd in different *Miscanthus* spp.

### Difference in Cd accumulation and transfer is relative to resistance among *Miscanthus* spp.

Gill et al. [6] reported that the uptake and transport of Cd differed with plant species and genotypes. Cd accumulation in leaves directly leads to damage to the photosynthetic apparatus and decreases in Pn [28,29]. The different concentrations of Cd in roots and leaves of *Miscanthus* spp., even for the same concentration of exogenous Cd treatment (Fig 2A and 2B) reflects the difference in absorption by roots and transport from roots to shoot, and explains the difference in resistance of *Miscanthus* spp. to Cd. The highest Cd concentration in roots (Fig 2A) and medium Cd concentration in leaves (Fig 2B) indicated restricted transport and more absorption for *M*. *sinensis*. The highest Cd concentrationin leaves (Fig 2B) and medium Cd concentration in roots (Fig 2A) suggested stronger transport and absorption for *M*. *floridulus*. The lowest Cd concentrations both in leaves and in roots confirmed much less absorption of exogenous Cd for *M*. *sacchariflorus* (Fig 2) and this low absorption is not only a characteristic but could be the main cause of the higher resistance of *M*. *sacchariflorus* to Cd.

### The decrease in Pn was due to lower activities of C_4_ photosynthetic enzymes and damage to chloroplast structure

Exogenous Cd treatment resulted in depression of Pn and AQY of all species, and the depression was much greater in *M*. *sinensis* and *M*. *floridulus* (Fig 3A and 3E). To determine the reason for this depression in *Miscanthus* spp. under different Cd concentrations, we determined the activities of key enzymes of the C_4_ photosynthetic pathway—PEPC, NADP-ME and NADP-MDH—that participate in the process of concentrating CO_2_ in C_4_ photosynthesis [38]. We found significant decreases (*p<0*.*05*) in PEPC, NADP-ME and NADP-MDH activity in all *Miscanthus* spp. exposed to Cd stress. However, PEPC activity was much higher in *M*. *sacchariflorus* than in the other two species for all Cd concentrations (Fig 5A). This was consistent with the highest Pn and higher Cd tolerance in *M*. *sacchariflorus*. The Pn of *Miscanthus* spp. were closely related to PEPC content rather than ribulose 1,5 bisphosphate carboxylase/oxygenase (Rubisco) under higher nitrogen content [36]. Moreover, in maize leaves it was found to be inactivated by Cd [39]. The NADP-ME is a key enzyme in the NADP-ME subtype of C_4_ plants and helps enrich the CO_2_ for Rubisco, thus lowering photorespiration and improving photosynthetic efficiency [40]. NADP-MDH is particularly abundant in C_4_ plants, where it functions photosynthetically in the NADP-dependent reduction of oxaloacetate to malate [41]. It was also reported that higher MDH activity and malate accumulation in companion with higher Pn were found in the drought-resistant *Sorghum bicolor* genotype compared with a sensitive genotype [42]. In the present study, the higher activity of NADP-MDH in *M*. *sacchariflorus* under Cd stress favored conversion of oxaloacetate to malate (Fig 5C) which is then transported into adjacent bundle sheath cells to enhance Calvin cycle in bundle sheath cells [43]. The increase in malate synthesis can cause a significant increase in root malate exudation, thus improving toxic metal resistance in C_3_ plants [44,45], but it is still unclear in C4 plant whether or not the malate, except for C_3_ CO_2_ fixation in leaf, can be transported from leaf to root. If a part of malate resulted from the higher activity of NADP-MDH in *M*. *sacchariflorus* under Cd stress can be transported out of leaf and reaches to root, it is possible to confer high Cd tolerance in this plants.

Chloroplast ultrastructure could provide important information concerning the biochemical properties of the thylakoids, which suffer the greatest changes during adverse environmental conditions, such as salt [46], drought [47] or heavy metal [48] stresses. The decrease of Pn is related to changes in the membrane structure of chloroplasts [49] and degradation of chloroplasts [48]. The production of starch grains, enlargement of osmiophilic globules and loss of the chloroplast envelope showed large differences among the *Miscanthus* species under various Cd concentrations (Fig 6D, 6G and 6J–6O). Cd caused the grana and stroma lamellae of chloroplasts to condense and the loss of connection between both lamellae in *M*. *sinensis* (Fig 6G, 6J and 6M) and *M*. *floridulus* (Fig 6N) but did not induce significant change in *M*. *sacchariflorus*. These results not only indicated the different resistance of *Miscanthus* spp. to Cd, but also confirmed that the different decreases in Pn, photosynthetic pigment, the maximal photochemical efficiency of PSII (Fv/Fm) and effective PSII quantum yield [Y(II)] (S3 Fig) resulted from damage to chloroplasts.

### Stronger anti-oxidant system may alleviate the damage to photosynthetic apparatus

Cd exposure initially results in severe oxidative stress, which in turn caused lipid peroxidation and H_2_O_2_ accumulation [9]. MDA is a product of lipid peroxidation and is considered an indicator of oxidative damage [50]. The present study showed that MDA accumulation increased most in *M*. *sinensis* under all Cd stress (Fig 7A), indicating that Cd induced stronger peroxidation and caused more serious damage to the cell membrane in *M*. *sinensis*. A certain amount of H_2_O_2_ accumulation during Cd stress may act as an oxidative agent and a local or systemic signal that activates various anti-oxidant enzymes, but over-accumulation of H_2_O_2_ induces peroxidative reactions that damage plant cells [51]. The SOD catalyzes the O_2_^–^ dismutation reaction to form H_2_O_2_. CAT and POD could catalyze H_2_O_2_ into water and oxygen, alleviating the oxidative damage caused by H_2_O_2_. However, in chloroplasts, H_2_O_2_ is restricted by the ascorbate–glutathione (ASH–GSH) cycle, where APX uses ASH as a hydrogen donor and GR catalyzes the NADPH-dependent reduction of oxidized glutathione (GSSG) to reduced GSH [52]. The activities of these enzymes were increased by Cd stress in wheat and tobacco [53,54]. In the present study, *M*. *sinensis* showed a greater increase in SOD activity (Fig 8A), but lesser increase in POD and APX activities (Fig 8C and 8D), resulting in greater H_2_O_2_ accumulation (Fig 7B) and the most serious damage to chloroplasts (Fig 6D, 6G, 6J and 6M), compared with the other *Miscanthus* spp. In *M*. *sacchariflorus*, for all concentrations of Cd treatment, the SOD, POD and APX activities were higher (Fig 8A, 8C and 8D) and the H_2_O_2_ accumulations much lower than that in the other *Miscanthus* spp. (Fig 7B). These results indicated that *M*. *sacchariflorus* possessed a better anti-oxidative system, which could scavenge ROS and maintain integrity of the chloroplast (Fig 6F, 6I, 6L and 6O). The results support the view that genotypic difference in the anti-oxidative system could partially account for the genotypic difference in Cd accumulation, tolerance and an increase in tolerance to Cd stress is positively correlated with anti-oxidant capacity [55].

## Conclusions

The present study revealed the effects of Cd on plant growth, photosynthesis characteristics, chloroplast ultrastructure, Cd-uptake and translocation and physiological responses of three *Miscanthus* species. The results showed that the effects of different Cd concentrations on growth and Pn in *Miscanthus* spp. differed. The inhibitory effect of Cd on growth characteristics was more evident for *M*. *sinensis* whereas, least for *M*. *sacchariflorus*. The resistance of *M*. *sacchariflorus* to Cd was mainly due to a lower Cd absorption and translocation, thus keeping more effective activities of C_4_ photosynthetic enzymes and better chloroplast structure. Furthermore, hyperactivity of anti-oxidant enzymes also played an important role in protecting *M*. *sacchariflorus* from Cd toxicity.

## Supporting Information

S1 Fig Change in net photosynthetic rate (Pn), stomata conductance (g_s_), intercellular CO_2_ concentration (Ci) with increasing PPFD under Cd stress for 16 days. (TIF)

S2 Fig The chloroplast ultrastructure in bundle sheath cells. Bar = 2μm.
(TIF)

S3 Fig Change in Chlorophyll fluorescence parameters, false-color images of maximal photochemical efficiency (Fv/Fm). (TIF)
