# Supplementary figures and images for: Different Growth and Physiological Responses to Cadmium of the Three Miscanthus Species
Source: PLoS One. 2016 Apr 12;11(4):e0153475. doi: 10.1371/journal.pone.0153475 (PMC4829268; doi:10.1371/journal.pone.0153475)

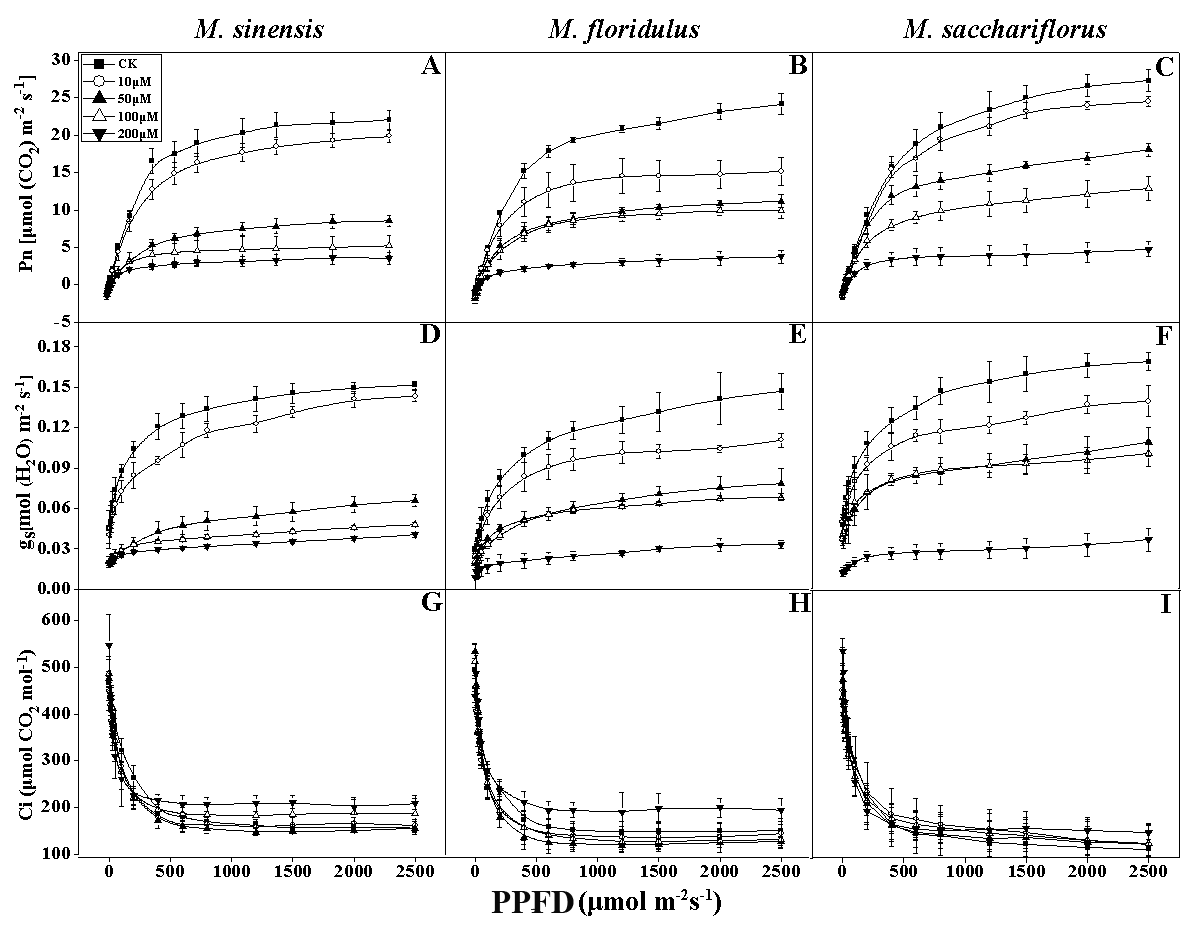

Supplement: S1 Fig — (TIF) [file pone.0153475.s001.tif]

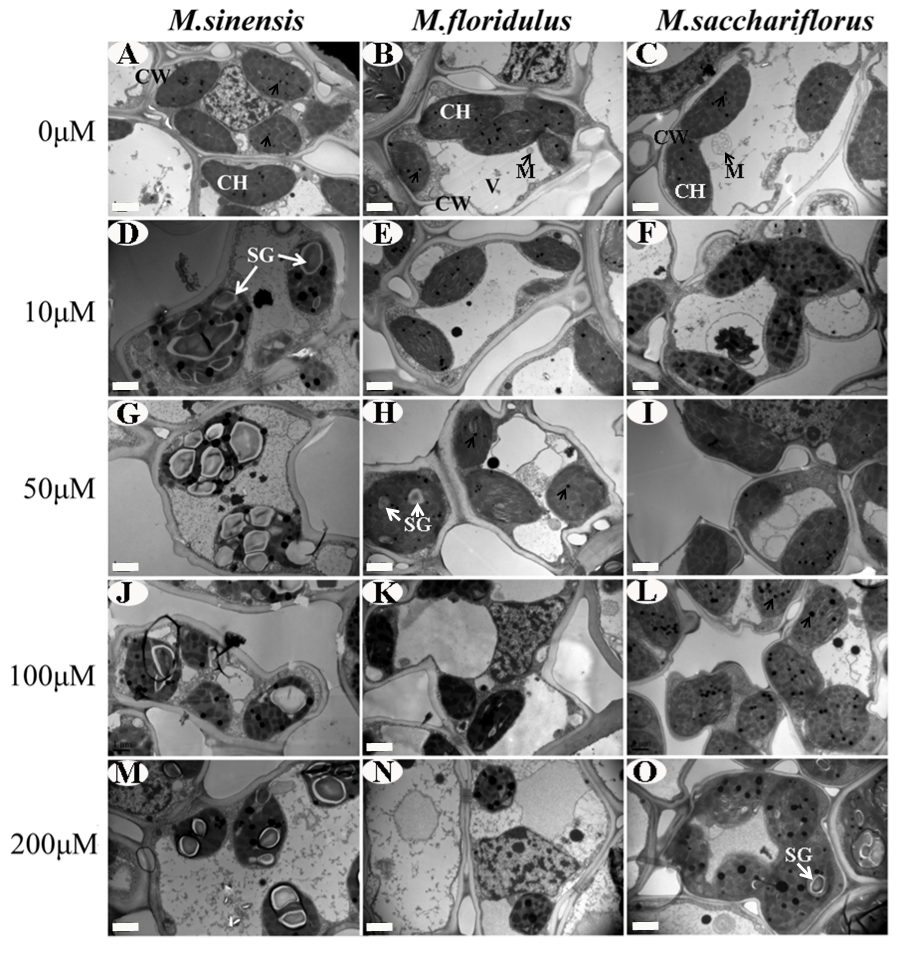

Supplement: S2 Fig — Bar = 2μm. (TIF) [file pone.0153475.s002.tif]

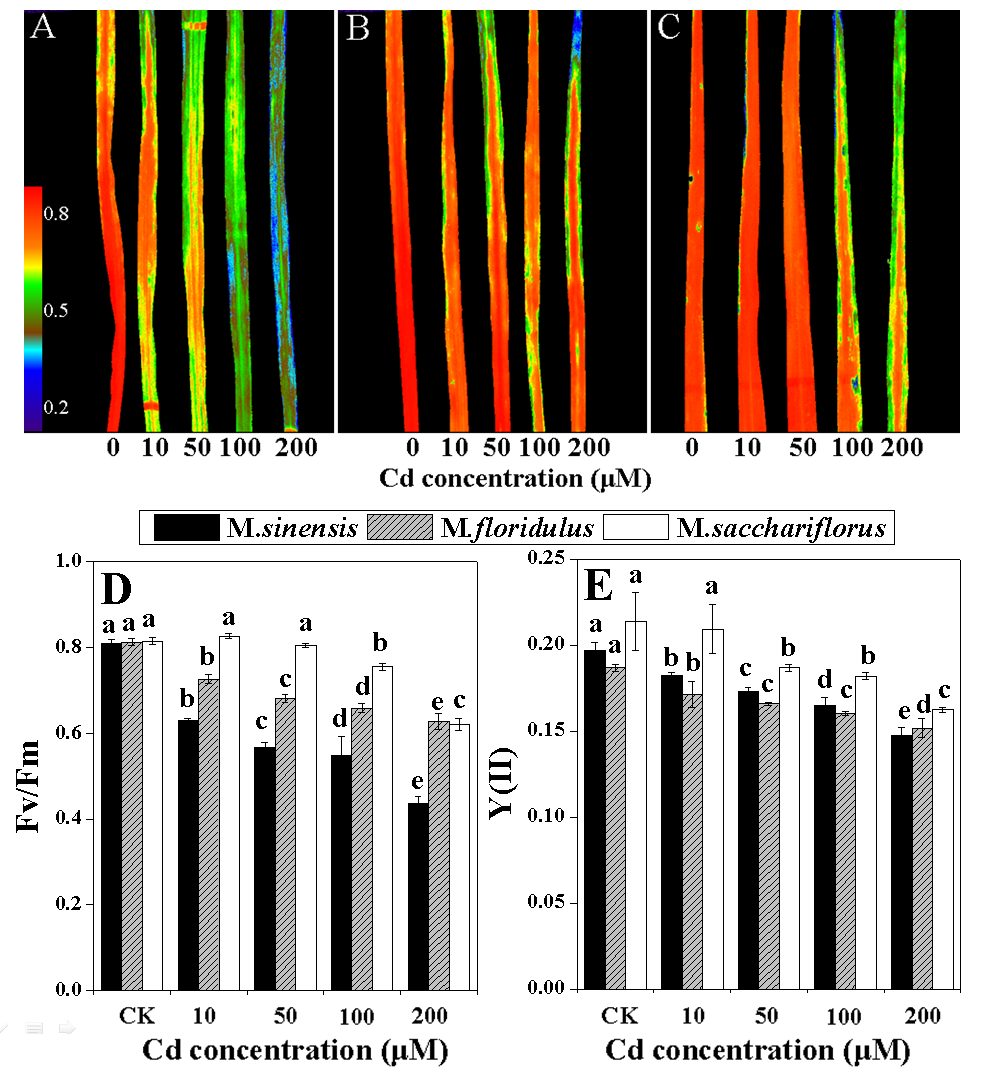

Supplement: S3 Fig — (TIF) [file pone.0153475.s003.tif]
